# Blood‐based markers of efficacy and resistance to cetuximab treatment in metastatic colorectal cancer: results from CALGB 80203 (Alliance)

**DOI:** 10.1002/cam4.806

**Published:** 2016-07-27

**Authors:** Ace J. Hatch, Alexander B. Sibley, Mark D. Starr, J. Chris Brady, Chen Jiang, Jingquan Jia, Daniel L. Bowers, Herbert Pang, Kouros Owzar, Donna Niedzwiecki, Federico Innocenti, Alan P. Venook, Herbert I. Hurwitz, Andrew B. Nixon, the Alliance for Clinical Trials in Oncology

**Affiliations:** ^1^ Duke University Medical CenterDurhamNorth Carolina; ^2^ Duke Cancer Institute Bioinformatics Shared ResourceDuke UniversityDurhamNorth Carolina; ^3^ Alliance Statistical and Data CenterDurhamNorth Carolina; ^4^ East Carolina UniversityGreenvilleNorth Carolina; ^5^ Department of Biostatistics and BioinformaticsDuke UniversityDurhamNorth Carolina; ^6^ School of Public HealthLi Ka Shing Faculty of MedicineThe University of Hong KongPok Fu LamHong Kong SARChina; ^7^ University of North CarolinaChapel HillNorth Carolina; ^8^ University of CaliforniaSan Francisco ‐ Helen Diller Family Comprehensive Cancer CenterSan FranciscoCalifornia

**Keywords:** Biomarker, cetuximab, colorectal cancer, plasma

## Abstract

Circulating protein markers were assessed in patients with colorectal cancer (CRC) treated with cetuximab in CALGB 80203 to identify prognostic and predictive biomarkers. Patients with locally advanced or metastatic CRC received FOLFOX or FOLFIRI chemotherapy (chemo) or chemo in combination with cetuximab. Baseline plasma samples from 152 patients were analyzed for six candidate markers [epidermal growth factor (EGF), heparin‐binding EGF (HBEGF), epidermal growth factor receptor (EGFR), HER2, HER3, and CD73]. Analyte levels were associated with survival endpoints using univariate Cox proportional hazards models. Predictive markers were identified using a treatment‐by‐marker interaction term in the Cox model. Plasma levels of EGF, HBEGF, HER3, and CD73 were prognostic for overall survival (OS) across all patients (*KRAS* mutant and wild‐type). High levels of EGF predicted for lack of OS benefit from cetuximab in *KRAS* wild‐type (WT) patients (chemo HR = 0.98, 95% CI = 0.74–1.29; chemo+cetuximab HR = 1.54, 95% CI = 1.05–2.25; interaction *P* = 0.045) and benefit from cetuximab in *KRAS* mutant patients (chemo HR = 1.72, 95% CI = 1.02–2.92; chemo+cetuximab HR = 0.90, 95% CI = 0.67–1.21; interaction *P* = 0.026). Across all patients, higher HER3 levels were associated with significant OS benefit from cetuximab treatment (chemo HR = 4.82, 95% CI = 1.68–13.84; chemo+cetuximab HR = 0.95, 95% CI = 0.31–2.95; interaction *P* = 0.046). CD73 was also identified as predictive of OS benefit in *KRAS* WT patients (chemo HR = 1.28, 95% CI = 0.88–1.84; chemo+cetuximab HR = 0.60, 95% CI = 0.32–1.13; interaction *P* = 0.049). Although these results are preliminary, and confirmatory studies are necessary before clinical application, the data suggest that HER3 and CD73 may play important roles in the biological response to cetuximab.

## Introduction

Colorectal cancer (CRC) is one of the most common cancers in both men and women, and remains one of the leading causes of cancer‐related death worldwide 1. Declining incidence rates and improvements in early detection and treatment have led to reduced overall mortality rates, but outcomes in patients with metastatic disease remain poor with an estimated 5‐year relative survival rate of approximately 12% 1, 2. Therapies targeting the activation and signaling of epidermal growth factor receptor (EGFR/HER1/ERBB1) have improved outcomes, but essentially all patients will develop treatment resistance and progress 3, 4. Improving outcomes in these patients is predicated on refining our understanding of the relationship between receptor expression and downstream signaling pathways.

EGFR is a member of the HER/ERBB family of receptor tyrosine kinases (RTKs) that also includes HER2/ERBB2, HER3/ERBB3, and HER4/ERBB4. Ligand binding to the extracellular domains of these receptors results in their homo‐ and hetero‐dimerization, leading to activation of their intracellular kinase domains 5. HER‐family RTKs are activated by several ligands, including epidermal growth factor (EGF) and heparin‐binding EGF (HBEGF), leading to differential activation of multiple downstream signaling pathways 6. Hetero‐dimerization between members of this RTK family provides specificity to the downstream signaling initiated by the ligands that bind these receptors, but also provides potential avenues for resistance to cetuximab, as well as other agents, that target the activity of a single member of this receptor family 7, 8.

Cetuximab is a monoclonal antibody that binds EGFR and competitively inhibits its interaction with EGF 9. Cetuximab is associated with improved clinical outcomes in metastatic CRC (mCRC) and advanced head and neck cancer 10, 11. The Cancer and Leukemia Group B (CALGB, now The Alliance for Clinical Trials in Oncology) 80203 trial was initiated to evaluate the efficacy of cetuximab as first‐line treatment of mCRC in combination with FOLFOX or FOLFIRI chemotherapy, but it was closed upon the approval of bevacizumab as first‐line treatment for mCRC in 2004 and its analysis plan was redesigned as a randomized phase II trial. CALGB 80405 was subsequently initiated as a head‐to‐head comparison of bevacizumab versus cetuximab in combination with FOLFOX or FOLFIRI chemotherapy. The clinical data of both trials have been previously reported 12, 13.

The search for predictive biomarkers for EGFR‐targeting therapies has included analyses of amplifications of the EGFR gene 14, amplifications of the hepatocyte growth factor receptor (cMET) 15, genome‐wide DNA methylation status 16, and both candidate‐based and unbiased surveys of gene transcription 17, 18, 19. Currently, activating mutations in the *RAS* genes (codons 12, 13, 61, 117, and 146 of *KRAS* and *NRAS*) or *BRAF* codon 600 (exon 15) are the only validated biomarkers of resistance to cetuximab and other EGFR‐targeting therapies in mCRC in widespread clinical use 20, 21, 22, 23. It is important to note that CALGB 80203 was initiated before the routine incorporation of *KRAS* mutational testing. These patients were retrospectively screened for mutations in codons 12 and 13, but they have not been analyzed using the more comprehensive *RAS* mutation screening that is now considered standard of care 24. Because patients with *KRAS* mutant (Mut) tumors are no longer treated with cetuximab this study provides access to a distinctive patient population. Recognizing the need to develop additional biomarkers that may predict for sensitivity and resistance to cetuximab, as well as prognostic markers that could guide the management of patients with mCRC, plasma and serum were collected at baseline during CALGB 80203.

Previously, we identified several prognostic and predictive biomarkers of benefit from cetuximab in patients enrolled in CALGB 80203 using mRNA isolated from formalin‐fixed paraffin‐embedded (FFPE) tumor tissue 17. That analysis indicated that *HER3* and *NT5E* (*CD73*) mRNA expression were predictive of benefit from cetuximab. In this report, we have continued our analysis of CALGB 80203 and assessed the levels of EGFR‐related proteins in plasma. Plasma levels of EGF, HBEGF, soluble EGFR, soluble HER2/ERBB2, soluble HER3/ERBB3, and soluble CD73 were quantified using multiplex ELISA‐based methods. Blood‐based biomarkers hold several advantages over fresh tumor biopsies, including reduced risks and costs, broader availability, and the ability to be monitored throughout the course of treatment. This is one of the first reports to identify prognostic and predictive blood‐based biomarkers from a randomized trial using cetuximab in the first‐line treatment of mCRC.

## Materials and Methods

### Sample collection

Peripheral venous blood was collected at baseline from consenting patients into vacutainers containing EDTA anticoagulant. Samples were centrifuged at 2500*g* for 15 min within 30 min of collection. Plasma was aliquoted, frozen in liquid nitrogen, and shipped to the CALGB (now part of the Alliance for Clinical Trials in Oncology) Pathology Coordinating Office for centralized storage. For these analyses, samples were shipped to our laboratory (Duke/Alliance Molecular Reference Lab) thawed on ice, realiquoted, and stored at −80°C prior to use.

### Study design and patients

Design details of the CALGB 80203 study have been previously described 12. Patients with previously untreated, advanced, or metastatic adenocarcinoma of the colon or rectum were assigned to FOLFIRI, FOLFIRI plus cetuximab, FOLFOX, or FOLFOX plus cetuximab treatment groups. This was a multicenter trial approved by the institutional review boards at each participating institution, and all the patients included in the analyses reported here provided consent. Of the patients who consented but were found to be ineligible, one patient did not have colorectal cancer and the other patient had no evaluable disease. This retrospective analysis conforms to the reporting guidelines established by the REMARK criteria.

### Plasma protein analysis

EGF, HBEGF, EGFR, and HER2 were analyzed using the Searchlight platform (Aushon Biosystems, Inc., Billerica, MA) following the manufacturer's protocol. Plasma samples were thawed on ice, centrifuged at 20,000*g* for 5 min, loaded onto SearchLight plates with standards, and incubated at room temperature for 1 h while shaking at 950 rpm (Lab‐Line Titer Plate Shaker, Model 4625, Barnstead, Dubuque, WI). All washing steps were performed using a plate washer (model ELx405; Biotek Instruments, Inc., Winooski, VT). After washing, biotinylated secondary antibody was added, and plates were incubated for 30 min, washed, streptavidin‐HRP was added, incubated for 30 min, and plates were washed again. SuperSignal substrate reagent was added after the final wash, images were collected within 10 min, and images were analyzed using SearchLight array analyst software.

HER3 and CD73 were analyzed using assays developed in our laboratory using the Meso Scale Discovery ELISA platform (Meso Scale Discovery, Rockville, MD). For HER3, ELISA plates were coated overnight with 4 *μ*g/mL HER3 capture antibody (MAB3481; R&D Systems, Minneapolis, MN). After sample incubation, HER3 was detected using 1 *μ*g/mL biotinylated HER3 antibody (MN BAF234; R&D Systems) and 5 *μ*g/mL streptavidin‐conjugated SulfoTag (R32AD‐5; Meso Scale Discovery). For CD73, ELISA plates were coated overnight with 3.3 *μ*g/mL CD73 capture antibody (550256; BD Biosciences, San Jose, CA). After sample incubation, CD73 was detected using 1 *μ*g/mL antibody (41‐0200; Invitrogen/Life Technologies, Grand Island, NY) conjugated to MSD Sulfo‐Tag according to the manufacturer's instructions (R91AN‐1, Meso Scale Discovery). Samples were quantified using MSD Discovery Workbench software version 3.0.18 (Meso Scale Discovery). All assays were performed in duplicate and laboratory personnel were blinded to clinical outcome.

### *KRAS* mutational analysis

*KRAS* mutation analysis was performed in the CALGB/Alliance molecular reference laboratory of Dr. Greg Tsongalis at Dartmouth Medical School using the TheraScreen KRAS Mutation Test Kit (870021; Qiagen, Manchester, UK).

### RNA Isolation

The isolation and quantification of mRNA transcripts using real‐time PCR was previously reported 17.

### Statistical analysis

Prognostic analyses were performed using baseline data from all available patients independent of treatment arm, with continuous values for the protein analytes. All marker levels were log‐transformed before analysis. Markers prognostic of clinical outcome (overall survival [OS] or progression‐free survival [PFS]) were determined using univariate Cox 29 proportional hazards models, and the resulting hazard ratios (HR), 95% confidence intervals (CI), and *P*‐values are reported. For each clinical outcome (OS or PFS) multivariate Cox regression models were used to test for interaction between marker level and treatment (chemo vs. chemo+cetuximab), to identify markers predictive of benefit from cetuximab. To further assess the role that *KRAS* mutational status has on subsequent biomarker determinations, the analyses were repeated for patients with *KRAS* wild‐type (WT) only and for *KRAS* Mut tumors only. Kendall's tau coefficient 26 was used to test for correlation between plasma protein levels and tumor mRNA expression for each marker using the subset of the analysis population for which both samples were available. *P*‐values were not adjusted for multiple testing.

Forest plots were created to depict the prognostic effect sizes (HRs and corresponding 95% CIs) of the different marker levels. For selected predictive markers, marker level was dichotomized at the median as “high” or “low”, and Kaplan–Meier 27 plots of OS or PFS were created with separate curves for each combination of treatment group and marker level.

The Alliance Statistics and Data Center conducted data collection and statistical analyses, and the clinical data were locked as of March 5, 2012. The R software environment for statistical computing and graphics 28 and the survival 25 package were used to execute the statistical analyses and to generate the figures.

## Results

### Patient characteristics

**Figure 1:**
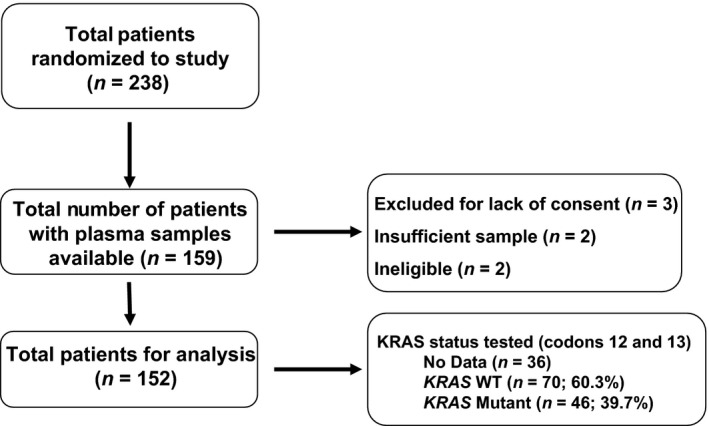
Study consort diagram.

The characteristics of the overall patient population were reported previously 12. Plasma samples were available for biomarker analysis from 152 of the 238 patients enrolled. The characteristics of this biomarker population reflected the characteristics of the overall study population (Table 1). As previously reported, there were no observed differences in outcomes between the groups that received FOLFOX or FOLFIRI chemotherapy, so these groups were combined into chemotherapy (chemo) alone (FOLFOX or FOLFIRI) and chemo+cetuximab groups for this analysis. No significant differences in the characteristics of the chemo and chemo+cetuximab groups were observed. *KRAS* mutational analysis was limited to the seven common mutations of the *KRAS* gene at codons 12 and 13. Extended *RAS* mutational analyses were not performed. *KRAS* mutational status was only available for 116 (76.3%) of the patients in this group. In the blood‐based biomarker cohort the rate of *KRAS* mutation is 39.7%, slightly less than the rate of 43.0% in the parent study and 46.6% in our previous analysis of mRNA expression from FFPE samples 12, 17. A CONSORT diagram is presented in Figure 1.

**Table 1:** Patient characteristics

	Chemo (*N* = 76)	Chemo+cetuximab (*N* = 76)	Total (*N* = 152)	*P*‐value
Age	Number (%Total)	Number (%Total)	Number (%Total)	0.24
20–29	2 (2.6)	1 (1.3)	3 (2.0)	
30–39	5 (6.6)	3 (3.9)	8 (5.3)	
40–49	7 (9.2)	13 (17.1)	20 (13.2)	
50–59	20 (26.3)	15 (19.7)	35 (23.0)	
60–69	29 (38.2)	22 (28.9)	51 (33.6)	
70+	13 (17.1)	22 (28.9)	35 (23.0)	
Gender				0.87
Male	47 (61.8)	49 (64.1)	96 (64.5)	
Female	29 (38.2)	27 (35.9)	56 (35.5)	
Race				0.33
White	64 (84.2)	69 (90.8)	133 (87.5)	
ECOG PS				0.19
0	34 (44.7)	43 (56.6)	77 (50.7)	
1	42 (55.3)	33 (43.4)	75 (49.3)	
KRAS status				1.00
Missing	20	16	36	
KRAS Mut	22 (39.3)	24 (40.0)	46 (39.7)	
KRAS WT	34 (60.7)	36 (60.0)	70 (60.3)	

### Biomarker characteristics

The six markers of interest were chosen based on their direct role in EGFR signaling, previous examination of mRNA levels in archived FFPE tumor samples, and the availability of high‐quality assays to accurately assess each soluble marker in patient plasma. The levels of EGFR markers in blood were measured and tested for association with both OS and PFS outcomes. The characteristics of the assayed markers are shown in Table S1. The EGFR ligands (EGF, HBEGF) were present at lower levels compared to the soluble receptors, and were observed to have higher levels of variability between patients. Baseline levels of the EGF and HBEGF ligands were positively correlated (Kendall rank correlation coefficient *τ *= 0.34), as were levels of HER2 and HER3 (*τ *= 0.33). No other marker pairs showed strong correlations (Table S2). Additionally, there was no association observed for any marker tested and *KRAS* mutation status (data not shown).

### Prognostic marker analyses

**Figure 2:**
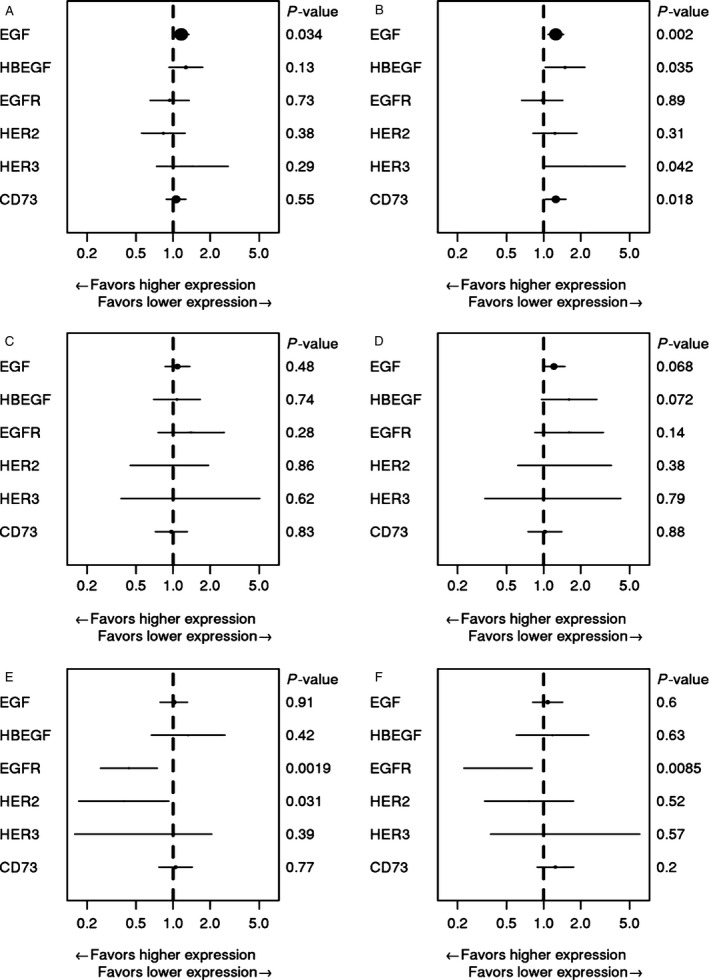
Prognostic forest plots showing the association of each marker with PFS (A, C, and E) or OS (B, D, and F) for all patients (A and B), *KRAS* WT patients (C and D), and *KRAS* Mut patients (E and F).

The prognostic association of protein levels with survival endpoints (PFS and OS) was examined in the overall population (Fig 2, panels A and B) as well as in the *KRAS* WT (Fig. 2, panels C and D) and *KRAS* Mut (Fig. 2, panels E and F) groups separately.

In the overall population, higher EGF protein levels were prognostic for shorter PFS (HR = 1.16, 95% CI = 1.01–1.34, *P* = 0.034) and OS (HR = 1.25, 95% CI = 1.09–1.45, *P* = 0.002) (Fig. 2, panels A and B). In the *KRAS* WT group, no association was observed between EGF and PFS (*P* = 0.482), but EGF levels showed a trend toward being prognostic for OS (HR = 1.21, 95% CI = 0.99–1.49, *P* = 0.068). In the *KRAS* Mut group, no prognostic associations were observed between EGF and PFS (*P* = 0.913) or OS (*P* = 0.596).

In the overall population, no prognostic association between HBEGF and PFS was observed (*P* = 0.13). For OS, higher HBEGF levels were prognostic in all patients for shorter OS (HR = 1.49, 95% CI = 1.03–2.16, *P* = 0.035). No prognostic association between HBEGF and PFS was observed in the *KRAS* WT group (*P* = 0.74). Higher HBEGF levels showed a trend toward being prognostic for shorter OS in *KRAS* WT patients (HR = 1.61, 95% CI = 0.96–2.69, *P* = 0.072). No prognostic associations were observed between HBEGF and PFS (*P* = 0.42) or OS (*P* = 0.63) in the *KRAS* Mut group.

EGFR is the direct molecular target of cetuximab and tumor levels of EGFR protein have been studied extensively as a potential predictive biomarker of cetuximab efficacy 29, 30, 31, 32. In the overall population of this study, and in the *KRAS* WT group, plasma levels of EGFR were not prognostic for PFS or OS. However, in *KRAS* Mut patients, higher EGFR levels were prognostic for both longer PFS (HR = 0.44, 95% CI = 0.26–0.74, *P* = 0.002) and longer OS (HR = 0.43, 95% CI = 0.23–0.80, *P* = 0.009).

In the overall population and in the *KRAS* WT group there were no prognostic associations observed between HER2 and PFS or OS. In *KRAS* Mut patients, higher HER2 was prognostic for longer PFS (HR = 0.40, 95% CI = 0.17–0.92, *P* = 0.031), but not OS (*P* = 0.52).

In the overall population, there was no prognostic association observed between levels of HER3 and PFS (*P* = 0.29). In the overall population, higher HER3 levels were prognostic for shorter OS (HR = 2.17, 95% CI = 1.03–4.58, *P* = 0.042). In the *KRAS* WT and *KRAS* Mut groups, there was no prognostic association observed between HER3 and PFS or OS.

CD73 has been implicated as a potential predictive biomarker for cetuximab in several reports, including our previous analysis of mRNA expression in FFPE samples from CALGB 80203 17, 18. In the current analysis, in the overall population higher plasma CD73 was prognostic for shorter OS (HR = 1.26, 95% CI = 1.04–1.52, *P* = 0.018). No additional prognostic associations between CD73 with PFS or OS were observed in the overall population or in the *KRAS* groups. All prognostic analyses for each marker are presented in Table S3.

### Predictive marker analyses

**Figure 3:**
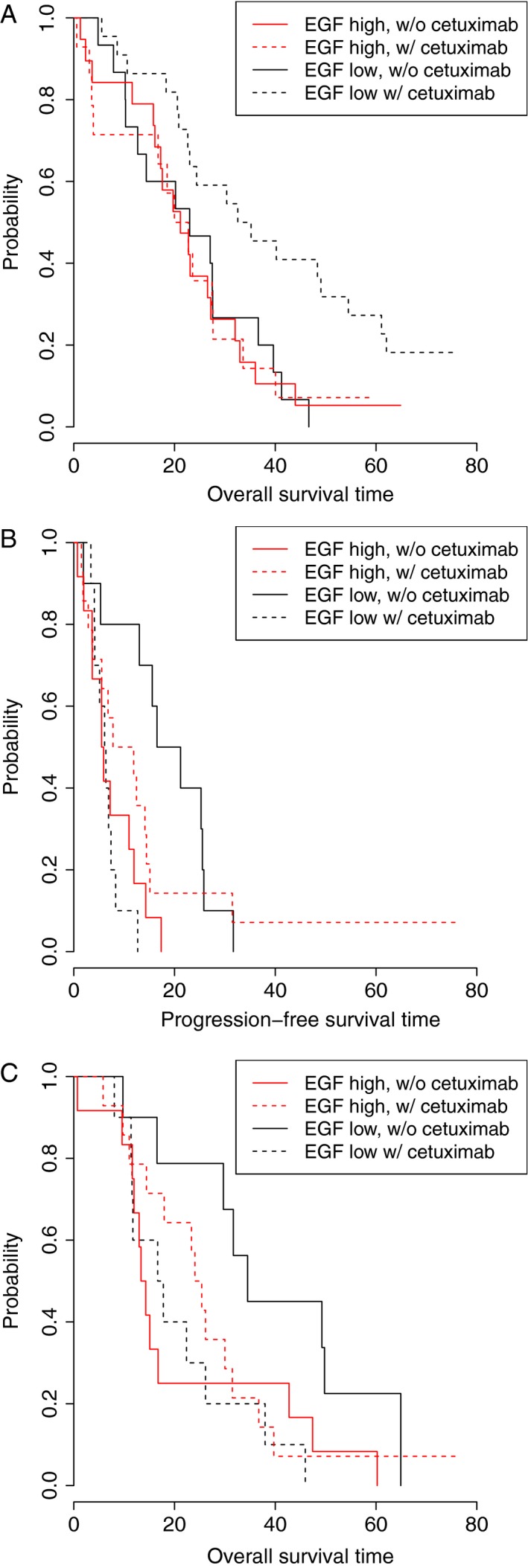
Kaplan–Meier curves showing the effects of EGF level and treatment arm. (A) OS *KRAS* WT patients (interaction *P* = 0.045); (B) PFS in *KRAS* Mut patients (interaction *P* = 0.001); (C) OS in *KRAS* Mut patients (interaction *P* = 0.026). High and low marker levels are dichotomized at the median. OS, overall survival; PFS, progression‐free survival; EGF, epidermal growth factor.

In the overall population, EGF protein levels were not predictive of PFS (interaction *P* = 0.233) or OS (interaction *P* = 0.748) benefit from cetuximab, but EGF levels were predictive within the individual *KRAS* groups. In the *KRAS* WT group, low EGF levels were predictive of OS benefit from cetuximab (chemo HR = 0.98, 95% CI = 0.74–1.29; chemo+cetuximab HR = 1.54, 95% CI = 1.05–2.25; interaction *P* = 0.045) (Fig. 3, panel A). In the *KRAS* Mut group, high EGF was predictive of benefit from cetuximab in both PFS (chemo HR = 2.16, 95% CI = 1.29–3.63; chemo+cetuximab HR = 0.76, 95% CI = 0.56–1.03; interaction *P* = 0.001) and OS (chemo HR = 1.72, 95% CI = 1.02–2.92; chemo+cetuximab HR = 0.90, 95% CI = 0.67–1.21; interaction *P* = 0.026) (Fig. 3, panels B and C).

Levels of HBEGF, EGFR, and HER2 were not predictive for either PFS or OS in the overall population or in either of the *KRAS* subgroups.

**Figure 4:**
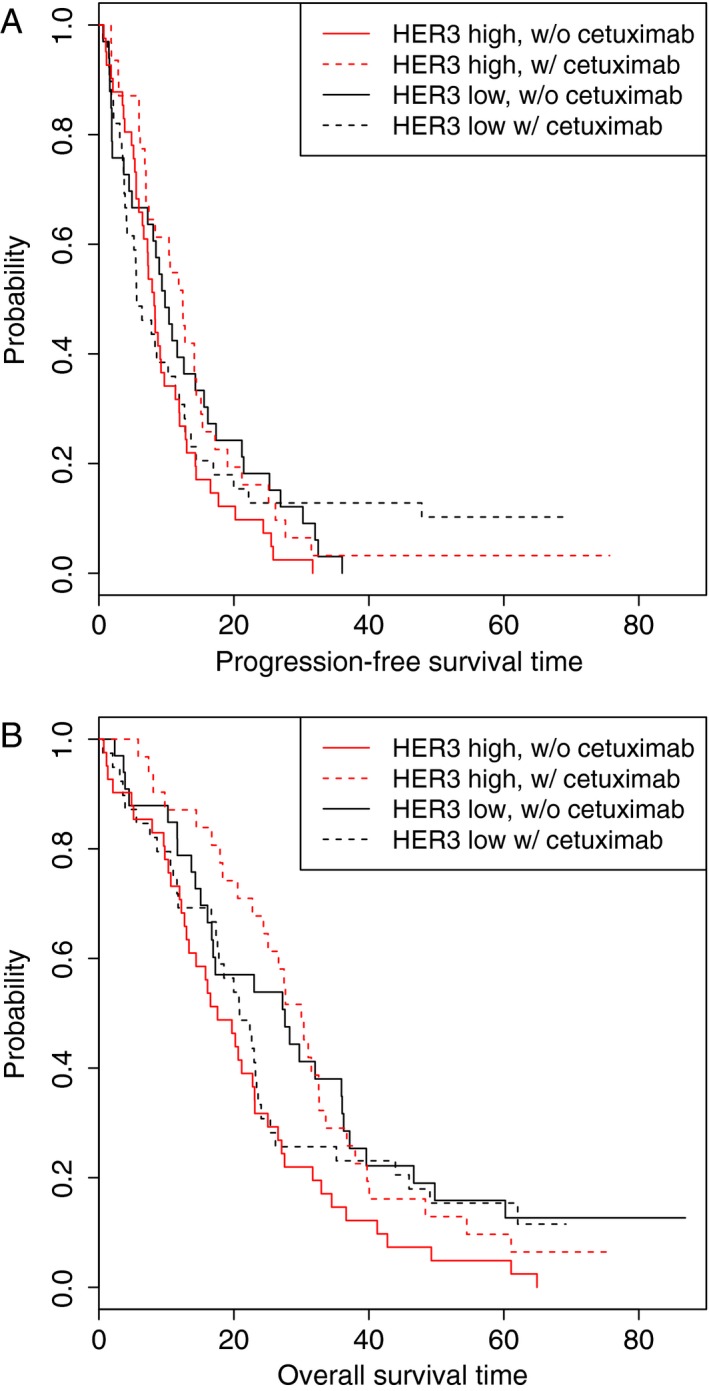
Kaplan–Meier curves showing the effects of HER3 level and treatment arm. (A) PFS in all patients (interaction *P* = 0.032); (B) OS in all patients (interaction *P* = 0.046). High and low marker levels are dichotomized at the median. OS, overall survival; PFS, progression‐free survival.

In the overall population, levels of HER3 were predictive for both PFS (chemo HR = 3.90, 95% CI = 1.41–10.80; chemo+cetuximab HR = 0.66, 95% CI = 0.25–1.78; interaction *P* = 0.032) and OS (chemo HR = 4.82, 95% CI = 1.68–13.84; chemo+cetuximab HR = 0.95, 95% CI = 0.31–2.95; interaction *P* = 0.046) (Fig. 4, panels A and B). It should be noted that the predictive role of HER3 was sensitive to the presence of an outlier with an extremely high level of plasma HER3. When this patient was removed from the analysis, HER3 was no longer predictive at *P* = 0.05, but the trends remained (PFS interaction *P* = 0.098, OS interaction *P* = 0.128). Interestingly, this patient did not have extreme values for any of the other markers examined, indicating that the high HER3 levels were unlikely due to preanalytic issues, such as sample handling, and may reflect the true levels of HER3 within this patient.

**Figure 5:**
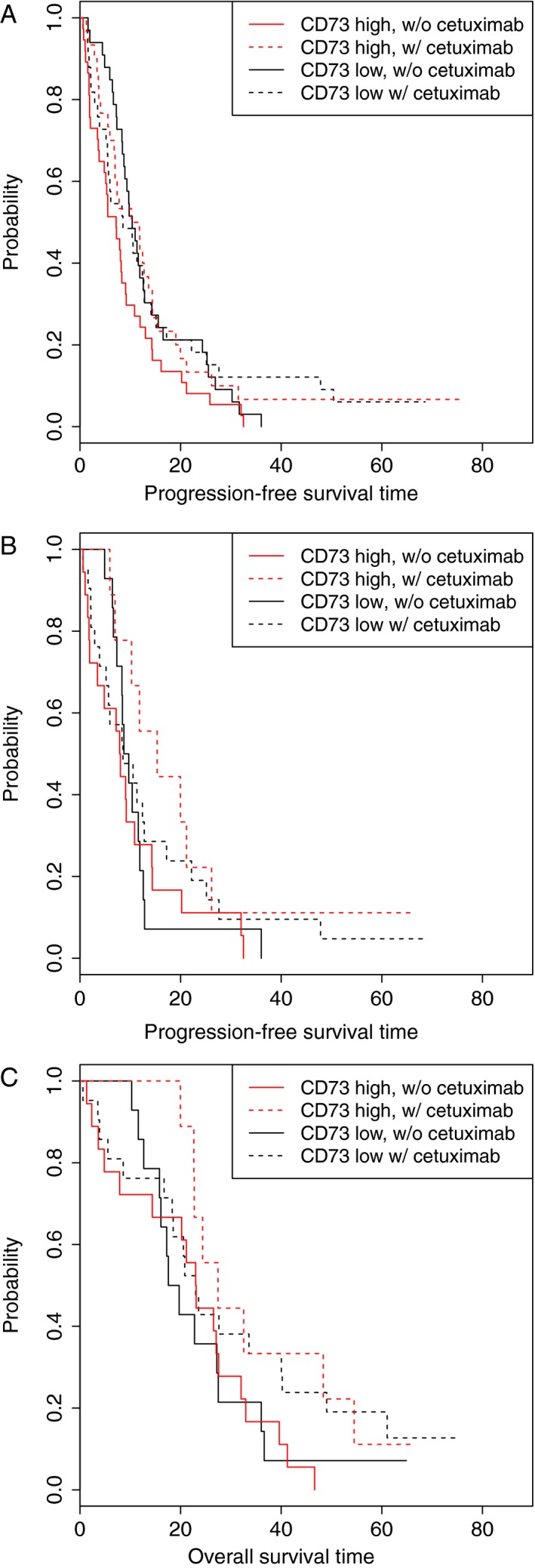
Kaplan–Meier curves showing the effects of CD73 level and treatment arm. (A) PFS in all patients (interaction *P* = 0.018); (B) PFS in *KRAS* WT patients (interaction *P* = 0.017); (C) OS in *KRAS* WT patients (interaction *P* = 0.049). High and low marker levels are dichotomized at the median. OS, overall survival; PFS, progression‐free survival.

In the overall population, CD73 levels were predictive of PFS benefit across all patients (chemo HR = 1.38, 95% CI = 1.08–1.77; chemo+cetuximab HR = 0.84, 95% CI = 0.63–1.12; interaction *P* = 0.018) (Fig. 5, panel A), but there was no predictive association observed between CD73 protein levels and OS. In the *KRAS* WT group, CD73 levels were predictive of PFS benefit from cetuximab (chemo HR = 1.32, 95% CI = 0.92–1.90; chemo+cetuximab HR = 0.61, 95% CI = 0.36–1.04; interaction *P* = 0.017) (Fig. 5, panel B) and OS benefit from cetuximab (chemo HR = 1.28, 95% CI = 0.88–1.84; chemo+cetuximab HR = 0.60, 95% CI = 0.32–1.13; interaction *P* = 0.049) (Fig. 5, panel C). No predictive effects for CD73 were observed in *KRAS* Mut patients.

### Comparison of plasma proteins and tumor mRNA expression

We previously identified several potential prognostic and predictive biomarkers from CALGB 80203 evaluating mRNA expression from FFPE tumor biopsies. In that work, we found that tumor expression of *HER3* and *CD73* were predictive biomarkers for cetuximab. The concordance between tumor‐based gene expression and plasma‐derived protein levels were explored. There were 71 patients who had both FFPE and plasma sample available for this concordance analysis. Across most samples, there was little association between tumor mRNA expression and plasma protein levels. EGF, HBEGF, EGFR, HER2, and CD73 exhibited no correlation between plasma protein levels and tumor mRNA expression levels. However, plasma HER3 protein and tumor *HER3* mRNA expression were modestly correlated with one another (*τ *= 0.22, *P* = 0.010).

## Discussion

Mutations in *KRAS*, and more recently *NRAS* and *BRAF,* are the only biomarkers regularly used for guiding the use of cetuximab in CRC and additional biomarkers are desperately needed. CALGB 80203 was initiated to evaluate cextuximab with FOLFOX or FOLFIRI chemotherapy in the first‐line setting, but was closed due to slow enrollment and the approval of bevacizumab as first‐line therapy for CRC. After closure of CALGB 80203, a new intergroup study was initiated to directly compare the efficacy of cetuximab versus bevacizumab in mCRC. In CALGB 80405 patients were randomized to receive standard chemotherapy with either bevacizumab (which targets vascular endothelial growth factor) or cetuximab in the first‐line setting. As reported at ASCO in 2014, CALGB 80405 did not identify any significant PFS or OS differences between the bevacizumab and cetuximab cohorts in *RAS* WT patients 33, 34. These results underscore the need to identify biomarkers beyond RAS that can select for patients who are most likely to benefit from cetuximab, as well as other targeted agents.

To identify biomarkers for cetuximab in mCRC, we assayed plasma levels of six proteins in patients that were either directly associated with the EGFR signaling pathway or previously implicated as potential biomarkers for cetuximab. Many of the evaluated proteins have been previously suggested as potential prognostic biomarkers; however, very few studies have evaluated the soluble levels of these proteins in patient plasma. While it is established that EGFR tumor levels are prognostic using immunohistochemical approaches 35, 36, other readouts, including *EGFR* mRNA expression and copy number, are less consistent 17, 37. In the overall population, plasma EGF was prognostic for both OS and PFS, contradicting other observations from serum measurements of colon cancer patients 38. However, differences could be due to the unknown *KRAS* status for patients in this earlier work or could reflect biological differences in plasma versus serum EGF levels. Interestingly, we observed that EGFR and HER2 were only prognostic in the *KRAS* Mut population. However, for HER3, we observed that levels were prognostic for OS across all patients, but when analyzed based on *KRAS* mutational status, no associations were observed. In fact, no markers were prognostic within the *KRAS* WT group. While the impact of *KRAS* mutations on soluble HER receptors and ligand levels and their role as prognostic factors remain unclear, no associations were observed between marker levels and *KRAS* mutation status in this study.

Protease‐mediated shedding is important for the processing of membrane‐associated ligands and has been implicated in the regulation of EGFR levels 39, 40, 41, 42. Because soluble EGFR is competent to bind EGF 41, high levels of soluble receptors may act as ligand sinks that downregulate signaling through the EGFR pathway by reducing both the amount of free ligand and the amount of cell‐surface receptors. Downregulation of HER3 protein on tumor cells is expected to improve outcomes from cetuximab therapy by reducing compensatory signaling through HER3‐containing heterodimers. Increased HER3 protein in patient plasma could reflect a process of active shedding as part of a homeostatic response to increased HER‐axis signaling that may play a role in tumorigenesis. Strategies inhibiting hetero‐dimerization between HER3 and other HER family receptors have been promising 43, 44, 45, and targeting HER3 directly in a preclinical model has been effective in overcoming acquired resistance to cetuximab 46.

This study provides data consistent with a model in which HER3 mediating resistance to cetuximab. HER3 shedding may suggest down‐regulation of this resistance pathway. In this study, plasma protein levels of HER3, measured by ELISA, and *HER3* mRNA from FFPE tumor tissue, measured using real‐time PCR, were found to be modestly correlated with each other (*τ *= 0.22, *P* = 0.010). However, these comparisons should be considered highly exploratory because not only were different methods used, tumor protein was not analyzed, and all tumor mRNA samples were isolated from the surgical resection of the primary tumor, which often occurred years before the collection of plasma on this trial.

CD73 is an extracellular 5' ectonucleotidase that functions with CD39 (ecto‐ATPase), adenosine kinase (AK; phosphorylation to form AMP), and adenosine deaminase (ADA; deamination to inosine) to convert proinflammatory extracellular ATP to anti‐inflammatory adenosine. The effects of extracellular adenosine on T‐cell function and the emerging role of CD73 and purinergic signaling in cancer immunotherapy have been reviewed elsewhere 47, 48, 49. Inhibition of CD73 enhances the effects of immune checkpoint inhibitors in a preclinical model further supporting a role for CD73 in suppression of antitumor immune responses 50. CD73 is expressed on lymphocytes and endothelial cells and mature CD73 is linked to the extracellular surface by a glycosyl phosphatidyl inositol anchor. The mechanism by which CD73 is released into the plasma remains to be studied, but higher levels of CD73 may reflect a mechanism of active shedding to regulate the immune‐modulatory effects of CD73 on lymphocytes and other immune cell populations.

While intriguing, there are several limitations to the findings of this study. While CALGB 80203 was randomized, the number of available samples was limited and the current biomarker analyses were developed retrospectively after completion of the study. The markers included in this study provide a cross section of factors related to EGFR/HER‐family signaling that includes both ligands and soluble receptors. Acknowledging the high number of factors capable of signaling through the EGFR/HER‐family of receptors, additional studies are required to comprehensively investigate the levels of all potential ligands as potential predictive biomarkers for EGFR‐targeting therapies in CRC. Lastly, there are several characteristics of CALGB 80203 that make this study unique, most interesting being that *KRAS* mutation status was not independently predictive of benefit from cetuximab. Additionally, the *P*‐values reported here have not been adjusted for multiple testing so conclusions must be considered preliminary and hypothesis generating.

In conclusion, we have identified several potential blood‐based, predictive, protein biomarkers of benefit from cetuximab in mCRC. Though these results should be considered preliminary, and further validation is required before any clinical application of these results, they provide further evidence supporting HER3‐targeting therapeutic strategies and implicate immune modulation as an important factor in the response to cetuximab. These results deserve further study and analyses of these markers in CALGB 80405 are currently ongoing.

## Clinical Trial.gov

A Phase II Trial Of Irinotecan/5‐FU/Leucovorin Or Oxaliplatin/5‐FU/Leucovorin With And Without Cetuximab (C225) For Patients With Untreated Metastatic Adenocarcinoma Of The Colon or Rectum (NCT: NCT00077233).

## Conflict of Interest

ABN has received research funding from Amgen (Inst), Pfizer (Inst), Incyte (Inst), and Tracon Pharmaceuticals (Inst); has received consultant/advisory compensation from Novartis, Pfizer, and Cerulean Pharma; and has pending patents. AP has received research funding from Bayer AG (Inst), Onyx Pharmaceuticals (Inst), Genentech (Inst), GlaxoSmithKline (Inst), Eli Lilly (Inst) and Bristol Meyers Squibb (Inst); and has received consultant/advisory compensation from Gilead Sciences. HIH has received research funding from Incyte (Inst), Genentech (Inst), Novartis (Inst), GlaxoSmithKline (Inst), and Tracon Pharmaceuticals (Inst); has received consultant/advisory compensation from Incyte, Novartis, Genentech, Bristol‐Myers Squibb, Eli Lilly, Amgen, Sanofi, Regeneron Pharmaceuticals, GlaxoSmithKline, Tracon Pharmaceuticals, Acceleron Pharma and Bayer AG; honoraria from Genentech and ImClone Systems; and has pending patents. AJH, ABS, CJ, and KO disclose pending patents related to this work. MDS, JCS, JJ, DLB, HP, DN, and FI declare that they have no conflicts of interest to disclose.

## Supporting information


**Table S1.** Marker properties.
**Table S2.** Kendall's Tau correlation coefficients for each plasma marker analyzed.
**Table S3.** Prognostic analyses for each marker.
