# Supplementary material for: Blood‐based markers of efficacy and resistance to cetuximab treatment in metastatic colorectal cancer: results from CALGB 80203 (Alliance)
Source: Cancer Med. 2016 Jul 27;5(9):2249–60. doi: 10.1002/cam4.806 (PMC5055181; doi:10.1002/cam4.806)
Supplement: Supplementary file 1 — Table S1. Marker properties. Table S2. Kendall's Tau correlation coefficients for each plasma marker analyzed. Table S3. Prognostic analyses for each marker. [file CAM4-5-2249-s001.docx]

**Supplemental Tables**

**Supplemental Table S1: Marker properties**

|  | **N** | **Units** | **Average** | **Median** | **Range** |
| --- | --- | --- | --- | --- | --- |
| **EGF** | 152 | pg/ml | 37.4 | 20.2 | 0.3-361.3 |
| **HBEGF** | 151 | pg/ml | 18.4 | 14.9 | 5.6-235.2 |
| **EGFR** | 152 | ng/ml | 26.1 | 25.7 | 3.5-49.4 |
| **HER2** | 152 | ng/ml | 3.7 | 3.3 | 1.4-25.1 |
| **HER3** | 144 | ng/ml | 11.6 | 11.0 | 6.6-45.8 |
| **CD73** | 133 | ng/ml | 25.9 | 12.8 | 2.7-202.3 |

Supplemental Table S2: Kendall's Tau correlation coefficients for each plasma marker analyzed

|  | **EGF** | **HBEGF** | **EGFR** | **HER2** | **HER3** | **CD73** |
| --- | --- | --- | --- | --- | --- | --- |
| **EGF** | 1 | 0.34 | -0.07 | 0.05 | 0.20 | 0.10 |
| **HBEGF** |  | 1 | -0.05 | 0.02 | 0.07 | 0.09 |
| **EGFR** |  |  | 1 | 0.13 | 0.05 | -0.02 |
| **HER2** |  |  |  | 1 | 0.33 | 0.23 |
| **HER3** |  |  |  |  | 1 | 0.25 |
| **CD73** |  |  |  |  |  | 1 |

**Supplemental Table S3: Prognostic analyses for each marker**

| **All Patients** | | | | | | |
| --- | --- | --- | --- | --- | --- | --- |
|  | **OS** | | | **PFS** | | |
| **Marker** | **HR** | **95% CI** | **p-value** | **HR** | **95% CI** | **p-value** |
| EGF | 1.25 | (1.09-1.45) | <0.01 | 1.16 | (1.01-1.34) | 0.03 |
| HBEGF | 1.49 | (1.03-2.16) | 0.04 | 1.27 | (0.93-1.74) | 0.13 |
| EGFR | 0.97 | (0.66-1.43) | 0.89 | 0.94 | (0.65-1.35) | 0.73 |
| HER2 | 1.24 | (0.82-1.86) | 0.31 | 0.83 | (0.56-1.25) | 0.38 |
| HER3 | 2.17 | (1.03-4.58) | 0.04 | 1.43 | (0.73-2.79) | 0.29 |
| CD73 | 1.25 | (1.04-1.52) | 0.02 | 1.06 | (0.88-1.27) | 0.55 |
| **KRAS WT** | | | | | | |
|  | **OS** | | | **PFS** | | |
| **Marker** | **HR** | **95% CI** | **p-value** | **HR** | **95% CI** | **p-value** |
| EGF | 1.21 | (0.99-1.49) | 0.07 | 1.09 | (0.86-1.37) | 0.48 |
| HBEGF | 1.61 | (0.96-2.69) | 0.07 | 1.08 | (0.70-1.66) | 0.74 |
| EGFR | 1.61 | (0.85-3.05) | 0.14 | 1.40 | (0.76-2.60) | 0.28 |
| HER2 | 1.48 | (0.62-3.54) | 0.38 | 0.93 | (0.45-1.94) | 0.86 |
| HER3 | 1.19 | (0.33-4.22) | 0.79 | 1.38 | (0.38-5.04) | 0.62 |
| CD73 | 1.03 | (0.75-1.40) | 0.88 | 0.97 | (0.72-1.30) | 0.83 |
| **KRAS Mut** | | | | | | |
|  | **OS** | | | **PFS** | | |
| **Marker** | **HR** | **95% CI** | **p-value** | **HR** | **95% CI** | **p-value** |
| EGF | 1.08 | (0.82-1.42) | 0.60 | 1.01 | (0.79-1.31) | 0.91 |
| HBEGF | 1.18 | (0.60-2.32) | 0.63 | 1.33 | (0.67-2.64) | 0.42 |
| EGFR | 0.43 | (0.23-0.80) | 0.01 | 0.44 | (0.26-0.74) | <0.01 |
| HER2 | 0.76 | (0.33-1.74) | 0.52 | 0.40 | (0.17-0.92) | 0.03 |
| HER3 | 1.50 | (0.37-6.02) | 0.57 | 0.57 | (0.16-2.06) | 0.39 |
| CD73 | 1.25 | (0.89-1.75) | 0.20 | 1.05 | (0.77-1.43) | 0.77 |
